# Lateral Piezoelectricity of Alzheimer's Aβ Aggregates

**DOI:** 10.1002/advs.202406678

**Published:** 2024-08-19

**Authors:** Jinhyeong Jang, Soyun Joo, Jiwon Yeom, Yonghan Jo, Jingshu Zhang, Seungbum Hong, Chan Beum Park

**Affiliations:** ^1^ Department of Materials Science and Engineering Korea Advanced Institute of Science and Technology (KAIST) 335 Science Road Daejeon 34141 Republic of Korea; ^2^ Applied Science Research Institute Korea Advanced Institute of Science and Technology (KAIST) 335 Science Road Daejeon 34141 Republic of Korea

**Keywords:** Alzheimer's disease (AD), beta‐amyloid, Kelvin probe force microscopy (KPFM), lateral piezoelectricity, vector piezoresponse force microscopy (PFM)

## Abstract

Alzheimer's disease (AD) is the most frequent neurodegenerative disorder in the elderly aged over 65. The extracellular accumulation of beta‐amyloid (Aβ) aggregates in the brain is considered as the major event worsening the AD symptoms, but its underlying reason has remained unclear. Here the piezoelectric characteristics of Aβ aggregates are revealed. The vector piezoresponse force microscopy (PFM) analysis results exhibit that Aβ fibrils have spiraling piezoelectric domains along the length and a lateral piezoelectric constant of 44.1 pC N^‐1^. Also, the continuous sideband Kelvin probe force microscopy (KPFM) images display that the increment of charge‐induced surface potential on a single Aβ fibril is allowed to reach above +1700 mV in response to applied forces. These findings shed light on the peculiar mechano‐electrical surface properties of pathological Aβ fibrils that exceed those of normal body components.

## Graphical abstract

This study unveils piezoelectric and electrical potential behaviors of Alzheimer's beta‐amyloid aggregates, as evidenced by different atomic force microscopy analyses. Beta‐amyloid aggregates demonstrate spiraling lateral piezoelectric domains, a notable piezoelectric constant of 44.1 pC N^‐1^, and surface potential exceeding +1700 mV under applied forces. These mechano‐electrical surface traits of beta‐amyloid aggregates suggest a correlation with neurodegenerative symptoms of Alzheimer's disease.

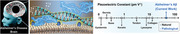

## 1 Introduction

Since Alois Alzheimer first described the deposition of sticky amyloid plaques in the brain of his dementia patient in 1906,^[^ ^1^ ^]^ the amyloid cascade hypothesis has elucidated the pathological mechanisms of Alzheimer's disease (AD) over the past century.^[^ ^2^, ^3^ ^]^ According to this hypothesis, the overproduction of beta‐amyloid (Aβ) peptides and the accumulation of their aggregates in the brain extracellular space are the initial events in different psychiatric symptoms of AD.^[^ ^4^, ^5^, ^6^, ^7^ ^]^ Aβ aggregation itself has remained an unanswered mystery, making the global AD community debate why Aβ peptides are overproduced in the brain,^[^ ^8^, ^9^ ^]^ how Aβ peptides self‐assemble into crystalline and thermodynamically‐stable Aβ fibrils,^[^ ^10^, ^11^ ^]^ and what is the physiological function of these Aβ fibrils.^[^ ^12^, ^13^ ^]^ Recent pathological test results have proposed that the accumulation of Aβ fibrils in the brain tissues triggers the hyperexcitability of the integral membrane proteins (e.g., mechanosensitive,^[^ ^14^, ^15^, ^16^ ^]^ voltage‐gated ion channels^[^ ^17^ ^]^), leading to different cell signaling pathways and apoptotic cell death as well.^[^ ^18^, ^19^, ^20^, ^21^, ^22^ ^]^ Although the structural details of Aβ fibrils have been reported using electron microscopes and nuclear magnetic resonance,^[^ ^23^, ^24^, ^25^, ^26^ ^]^ their nanoscopic surface properties disturbing the biological equilibrium remain unclear. In particular, it has been a challenge to answer why the direct contacts between Aβ fibrils and plasma membranes attenuate the biophysicochemical properties of brain cells, which are constantly subject to electrical communication and intracranial pressure.

**Figure 1:**
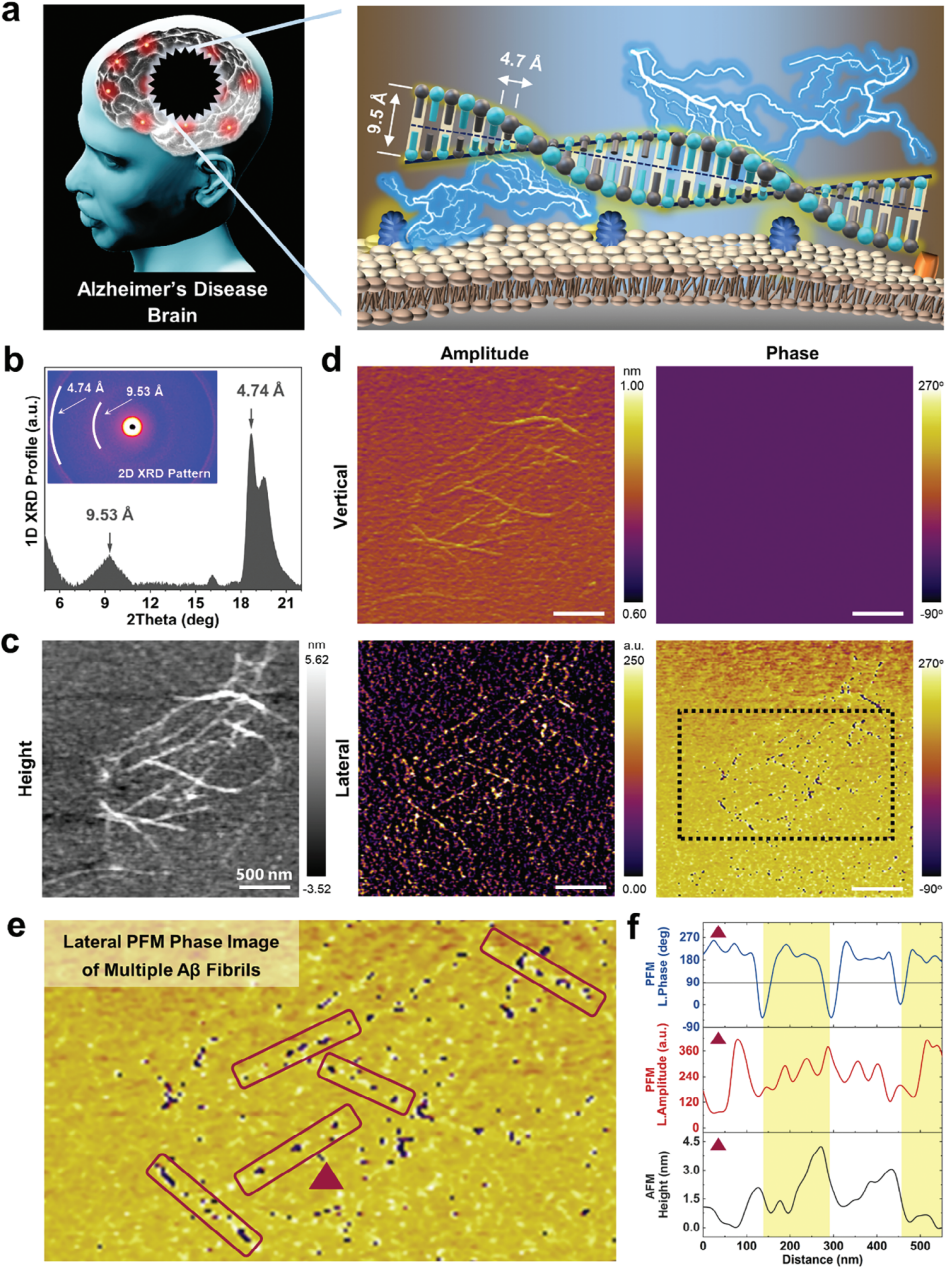
Crystalline Aβ fibril and its vector PFM images. a) Schematic illustration of Aβ fibrils with the piezoelectric nature. In the extracellular matrix of early AD brains, multiple monomeric Aβ peptides make a crystalline Aβ fibril by forming dimers with the distance of 9.5 Å and by stacking the dimers with the distance of 4.7 Å. The accumulation of these crystalline Aβ fibrils on brain cells is known to cause hyperexcitability of ion channels and neurodegeneration, leading to the multiple clinical symptoms of AD. However, the surface properties of Aβ fibrils involved in these events remain a mystery. This study unveils the giant piezoelectric nature of Aβ fibrils, which are constantly influenced by numerous electrical signals and fluid flows in the brain extracellular matrix. b) X‐ray diffraction (XRD) analysis results of prepared Aβ fibrils. 2D XRD pattern and its 1D profile represent that Aβ fibrils have interplanar spacing at 4.74 and 9.53 Å with crystallinity. c) Atomic force microscopy (AFM) topography, d) vertical and lateral piezoresponse force microscopy (PFM) images of crystalline Aβ fibrils. Black dot box in lateral PFM phase images shows e) magnified area. Red boxes mark individual Aβ fibrils, identified from topography, and a red triangle indicates a representative single Aβ fibril. f) Profiles of the representative single Aβ fibril with its lateral PFM phase, lateral PFM amplitude, and topography height. Each profile displays information for a single line without averaging for multiple pixels. Additional profiles of other Aβ fibrils marked with red boxes are in Supporting Information.

Here we report the orientation‐dependent electromechanical surface characteristics Aβ fibrils. Following the first discovery of the piezoelectric effect by the Curie brothers using a quartz crystal in 1880,^[^ ^27^ ^]^ the exploration of piezoelectricity has propelled rapid advancements in both uncovering and developing piezoelectric materials. Piezoelectric properties in a crystalline material stem from simultaneous displacements of electric and elastic dipoles, allowing the conversion of an applied mechanical pressure into an electric field emission and vice versa.^[^ ^28^ ^]^ Unlike piezoelectric studies aimed toward engineering mass‐producible composites for applications in energy and electronics (e.g., energy harvesters, sensors, transducers),^[^ ^29^ ^]^ the inherent piezoelectric behavior of biological components (e.g., collagen,^[^ ^30^, ^31^ ^]^ bone,^[^ ^32^, ^33^ ^]^ insulin protein,^[^ ^34^ ^]^ di‐ and tri‐peptide assemblies,^[^ ^35^, ^36^ ^]^ single amino acid crystals^[^ ^37^, ^38^ ^]^) has been explored to understand the physiological effects of applied mechanical pressure.^[^ ^39^, ^40^ ^]^ E.g., biomechanical stresses placed on bone tissue induce charge separation and subsequent neutralization processes that alter the chemistry of surrounding proteins and cells, aiding its growth and remodeling.^[^ ^41^ ^]^ The intrinsic piezoelectricity of different biological tissues primarily originates from bone minerals (hydroxyapatites^[^ ^42^ ^]^) and collagen fibers that comprise the extracellular matrix with a highly‐aligned structure.^[^ ^30^, ^39^ ^]^ While it is conceivable that the functionality of the extracellular matrix may be compromised by piezoelectric substances beyond collagen, the potential piezoelectricity of different pathological causes remains enigmatic. As depicted in **Figure** **1**a, we have presented the hidden lateral piezoelectric characteristics of Aβ fibrils generating large amounts of electric charge and surface potential in response to applied mechanical stimuli, compared to normal body components. Our findings unveil the capability of Aβ fibrils to convert surrounding bio‐mechanical stimuli into intense localized electric fields. This study provides an important hint that the induced neuronal disturbances relevant to the AD symptoms may arise from the continuous piezoelectric impact of Aβ fibrils, which accumulate in most brain regions at the preclinical stage.

## 2 Results

### 2.1 Crystalline Aβ Fibrils and Their Vector PFM Images

We prepared Aβ fibrils in an aqueous environment by self‐assembling monomeric Aβ_(1‐42)_ peptides, the most neurotoxic Aβ variant closely linked to AD. As‐prepared Aβ fibrils exhibited a typical fibril morphology with an axial length of micrometers and a radial thickness of 5 to 10 nm, and formed a network structure (Figure S1, Supporting Information), which is in accordance with those found in AD brains.^[^ ^43^ ^]^ Our X‐ray diffraction analysis results verified the crystallinity of Aβ fibrils with the intermolecular distances at 4.74 and 9.53 Å (Figure 1b). These values correspond to the revealed structural characteristics of Aβ fibrils, in which Aβ dimers with an intermolecular distance of 9 to 10 Å are helically stacked with a spacing of 4.7 Å to form a cross β‐sheet structure with 2_1_ screw symmetry.^[^ ^25^, ^44^ ^]^ Also, our circular dichroism analysis results confirmed the presence of the dominant cross β‐sheet secondary structure in Aβ fibrils, distinct from the amorphous structure of individual Aβ monomers (Figure S2, Supporting Information).

We hypothesized that crystalline Aβ fibrils may have peculiar piezoelectric properties based on the crystalline structure of the self‐assembled peptides and the hyperexcitability of the plasma membrane proteins in AD brain tissues. To unveil piezoelectric properties of Aβ fibrils, we employed a vector PFM technique on topologically‐distinguishable individual Aβ fibrils (Figure 1c, Experimental Section). Vector PFM is a type of atomic force microscopy (AFM) that allows simultaneous probing of the lateral (i.e., in‐plane) and vertical (i.e., out‐of‐plane) displacements induced by applying a bias to a nanoscopic region of an object.^[^ ^45^, ^46^ ^]^ To achieve enhanced spatial resolution in our measurements, we employed an AFM cantilever tip with a radius of curvature less than 25 nm and a shape of polygon‐based pyramid. These parameter closely aligns with those documented in prior research investigating piezoelectric properties within non‐pathological nanoscale biological entities, which employed tips featuring radii of curvature of 42 nm or below and different shapes (e.g., tetrahedral, square pyramidal) (Table S1, Supporting Information).^[^ ^30^, ^31^, ^32^, ^33^, ^34^, ^35^, ^36^, ^37^, ^38^ ^]^ As shown in the vector PFM images (Figure 1d; Figure S3, Supporting Information), the vertical piezoelectric phase of Aβ fibrils was indistinguishable from the background ranging from −90° to 270°. This result implies that the observed vertical piezoelectric components are caused by the shot noise and the cross‐talk of Aβ fibril topography,^[^ ^47^ ^]^ thus Aβ fibrils have negligible out‐of‐plane piezoelectric properties in the nanoscopic level. In contrast, both lateral piezoelectric amplitude and phase images of Aβ fibrils indicate distinct in‐plane piezoelectric properties of Aβ fibrils. Our magnified lateral piezoelectric phase image of Aβ fibrils shows that the complete iterative inversion of lateral piezoelectric phase occurs at intervals of 50 to 150 nm only in regions where Aβ fibrils are present (Figure 1e). It is consistent with the helical structure of Aβ fibrils with the crossover distance from 50 to 150 nm.^[^ ^23^, ^24^, ^25^, ^26^ ^]^ Also, we found that the representative lateral phase profiles of Aβ fibrils showed little correlation with their height profiles (Figure 1f; Figure S4, Supporting Information) at multiple different sites, suggesting that the lateral piezoelectric signals were derived from the nature of Aβ fibrils rather than the shot noise and topographic cross‐talk. Furthermore, the absence of artifacts is further supported by the matching trace and retrace of Aβ fibril's lateral piezoelectric images taken at two different angles (Figure S5, Supporting Information). Thus, our vector PFM images of Aβ fibrils were obtained absent of both deformation and charge noise artifacts, which is consistent with our theoretical calculations based on the Hertzian model and the Coulomb's law (Supporting Calculations 1 and 2).

### 2.2 Lateral Piezoelectric Characteristics of Aβ Fibrils

**Figure 2:**
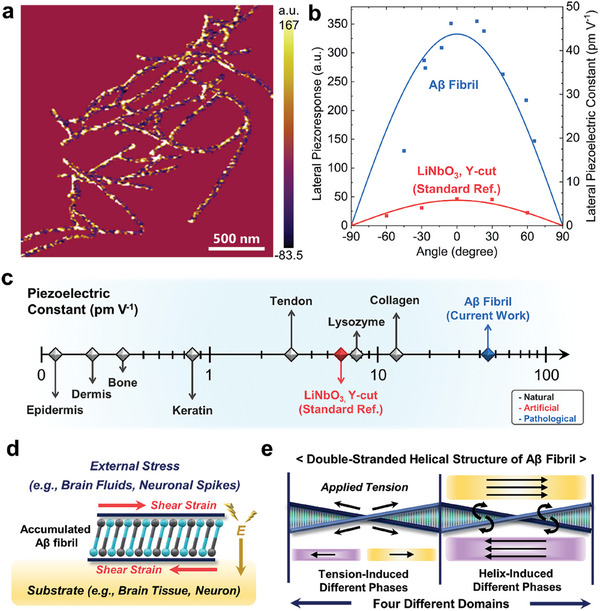
Structure‐derived lateral piezoelectricity of Aβ fibril. a) Lateral piezoresponse image and b) angle‐dependent magnitude of Aβ fibrils. To analyze the lateral piezoelectric constant of Aβ fibrils, Y‐cut LiNbO_3_ crystal was employed as standard reference. c) Comparison of piezoelectric constants of different body components with Aβ fibrils. The piezoelectric constants of body components were referred to the literature.^[^ ^40^ ^]^ Note that the inverse piezoelectric units of pm V^‐1^ are thermodynamically equiv. to direct piezoelectric units of pC N^‐1^. Schematic illustrations of d) the lateral piezoelectric effect of an accumulated Aβ fibril on a substrate and e) four different lateral piezoelectric domains based on the double‐stranded helical structure of Aβ fibril.

To evaluate the lateral piezoelectric constant of Aβ fibrils, we obtained their lateral piezoresponse images by combining the lateral piezoelectric amplitude and phase with the following Equation (1). As shown in **Figure** **2**a, Aβ fibrils (n = 10) displayed dotted‐like patterns in their lateral piezoresponse because of the effect of the lateral piezoelectric phases. Also, each Aβ fibril exhibited different maximum lateral piezoresponse magnitude that varied sinusoidally with its alignment orientation angles on the substrate (Figure 2b; Figure S6, Supporting Information). This variation of the lateral piezoresponse magnitudes is attributed to the cantilever geometry adopted for PFM scanning, where a subject should exhibit a maximum shear response when its longitudinal axis is perpendicular to the cantilever axis.^[^ ^45^ ^]^ We analyzed the maximum lateral piezoresponse magnitude of each Aβ fibril with its orientation angle compared to that of Y‐cut lithium niobate (LiNbO_3_), a standard piezoelectric reference material. As displayed in the calibration curves (Figure 2b), Aβ fibril exhibits giant lateral piezoelectric constant of 44.1 pC N^‐1^. According to the literature,^[^ ^40^ ^]^ most human tissues show very weak piezoelectric constants less than 2.0 pC N^‐1^ (e.g., dermis: 0.1 pC N^‐1^, bone: 0.2 pC N^‐1^, tendon: 2 pC N^‐1^). These values are derived from the dominant collagen fibers, which possess a piezoelectric constant ranging from ≈12.0 pC N^‐1^ to as low as 0.31 pC N^‐1^ in an ideal crystalline form (Figure 2c; Table S2, Supporting Information).^[^ ^40^, ^48^ ^]^ Our analysis result suggests that accumulated Aβ fibrils locally generate piezoelectric charges at least 3.6 times greater than the normal extracellular matrix in response to applied bio‐mechanical force (Figure 2d).

(1) $$\mathrm{Piezoresponse}=\mathrm{Amplitude}\times \cos\left(\mathrm{Phase}\right)$$

Moreover, Aβ fibrils displayed mottled patterns of lateral piezoelectric response on their surface (Figure 2a). This is attributed to different strain modes occurring simultaneously in the self‐assembled and helical structured Aβ fibrils under applied stress. From the presence of tensile strain (between self‐assembled Aβ peptide units) and torsional strain (within their helical structure) on the surface of Aβ fibrils (Figure 2e), we postulated that Aβ fibrils contain four independent lateral piezoelectric domain groups with different vector behavior along their longitudinal axis.

### 2.3 Distribution of Lateral Piezoelectric Vectors on the Surface of Aβ Fibrils

**Figure 3:**
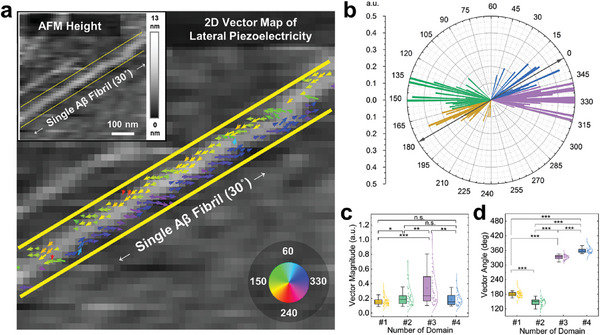
Lateral piezoelectric vectors on single Aβ fibril. a) Lateral piezoelectric vector map image of single Aβ fibril. Arrow color and length show the direction and magnitude of lateral piezoelectric vectors, respectively. Inset demonstrates topography of single Aβ fibril. Yellow lines in the images represent the area of the single Aβ fibril. b) Polar plot of lateral piezoelectric vectors of the Aβ fibril. Radii represent the normalized magnitude of lateral piezoelectric vectors. Four different colors denote different lateral piezoelectric domains of the Aβ fibril classified by k‐means clustering. c) Magnitude and d) angle distributions of lateral piezoelectric vectors of the single Aβ fibril in each domain. Plotted lines in each domain represent the fitted gamma distribution. All data were statistically analyzed by means of one‐way analysis of variance (ANOVA) (*p < 0.05; **p < 0.01; ***p < 0.001; n.s., not significant).

To reveal the presence of different lateral piezoelectric domain groups in a single Aβ fibril, we constructed a two‐dimensional vector map by superimposing two lateral piezoresponse images taken with 90° difference at the same spot. These two lateral piezoresponse images were utilized to reconstruct the in‐plane orientation of each pixel's piezoresponse, which varies sinusoidally with respect to the angle between the object and the probe axis.^[^ ^45^ ^]^ Note that a single lateral piezoresponse image would provide only partial orientation information on the surface of an Aβ fibril, which exhibits a helically self‐assembled structure with 2_1_ screw symmetry.^[^ ^25^, ^44^ ^]^ The obtained lateral piezoresponse vectors are overlapped on the grayscale AFM topography image of a single Aβ fibril with different colors representing their vector directions (**Figure** **3**a). The result shows that the magnitude of the lateral piezoresponse vectors was negligible at the central region of the Aβ fibril along the longitudinal axis. In contrast, two edges of the Aβ fibril had distinct lateral piezoresponse vectors with completely opposite angles. Also, each edge of the single Aβ fibril exhibited two lateral piezoelectric response vector groups having angles of ≈0 and 30° relative to its longitudinal axis respectively, and the two groups occurred repeatedly with the interval of ≈50 to 100 nm. Collectively, the lateral piezoelectric response vectors of the single Aβ fibril were shown to have four groups forming a spiral pattern along the edges.

We adopted *K*‐means clustering to the lateral piezoelectric vector components on the surface of the single Aβ fibril. *K*‐means clustering is a classical partition‐based algorithm to cluster large datasets by minimizing the sum of squared distances between each cluster.^[^ ^49^ ^]^ According to our computational analysis results (Figure 3b), the lateral piezoelectric vector components on the surface of the single Aβ fibril were clearly classified into four groups and the adjacent two groups had 28.5° and 34.5° as average differences against the orientation angle of the single Aβ fibril, respectively. These analyzed values were consistent with the vector map image, which visually displays the angle differences between two adjacent vector groups of ≈30° against the orientation angle of the single Aβ fibril. Also, we found negligible vector population on the single Aβ fibril in the two angle regions encompassing 90° and 270°, which are perpendicular to its longitudinal axis. The population of vector directions strengthens the lateral piezoelectric characteristics of Aβ fibril confined along the axial direction. Additionally, the magnitudes and angles of each lateral piezoelectric vector group are approximated by gamma distributions (Figure 3c,d).

### 2.4 Surface Potential Properties of Aβ Fibrils

**Figure 4:**
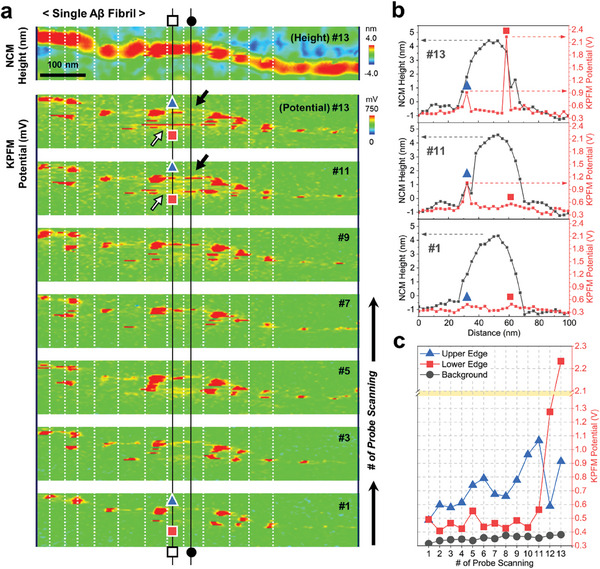
Surface potential changes on single Aβ fibril under repetitively‐applied electrostatic pressures. a) Continuous KPFM images of a single Aβ fibril at different scanning numbers. A representative non‐contact mode (NCM) AFM topography image of the single Aβ fibril with the same spot as the KFPM image is shown to indicate the sites of accumulated surface charge. The number of KPFM scanning was marked on the upper right. Vertical black line marked with white square boxes in KPFM images displays the sites of the single Aβ fibril for b) the representative potential and height profiles and c) surface potential changes following the increment of scanning numbers. The background surface potential in KPFM images was derived from the work function of Au/Cr substrate. Blue triangle and red square in the KPFM images mark two peak sites, respectively. White and black arrows in KPFM images of the single Aβ fibril indicate the sites of surface charge accumulation and release, respectively. Vertical black line marked with black circles in KPFM images provides the sites of the additional potential and height profiles of the single Aβ fibril, which are displayed in Supporting Information.

We hypothesized that the two edges of Aβ fibrils may exhibit larger surface potential than normal biological components due to the accumulation of piezoelectrically‐generated charges in response to external stress. To investigate the surface potential changes in an Aβ fibril, we employed sideband Kelvin probe force microscopy (KPFM), a technique based on frequency modulation (FM) principles. Unlike amplitude modulation (AM) KPFM (i.e., typical KPFM), which relies on detecting electrostatic forces, FM KPFM detects force gradients. This distinction is crucial as FM KPFM allows for the measurement of local interactions specifically at the tip apex, resulting in at least ten times stronger contrast of different surface components compared to AM KPFM.^[^ ^50^, ^51^ ^]^ Note that even a typical KPFM technique is able to be employed for analyzing voltage distributions along a thin nanowire with the radius of 7 nm.^[^ ^52^, ^53^, ^54^ ^]^ We captured multiple consecutive KPFM images of a single Aβ fibril on a gold/chromium (Au/Cr) substrate to analyze the surface potential changes when the electrostatic force of the cantilever probe tip was repetitively applied to the soft and elastic organic matter (**Figure** **4**a), which has the Young's modulus of 3.2 GPa close to that of human bone.^[^ ^55^ ^]^ As displayed in the initial KPFM image (scan #1), the surface charge values at most edge regions and the central region of the single Aβ fibril were similar to that of the background showing ≈300 mV. This result is attributed to the work function difference between the cantilever probe tip (4.9 to 5.4 eV) and the Au/Cr substrate (4.5 to 5.1 eV), indicating none‐to‐minimal accumulated surface charges on most regions of the single Aβ fibril. Interestingly, only a few edge regions of the single Aβ fibril possessed the distinct surface potential above 700 mV in the initial KPFM image. This distinct surface potential is derived from the electrostatic pressure and the strain‐induced charges on the piezoelectric Aβ fibril applied by the cantilever probe tip. In line with our speculation, we observed increasing surface potential of empty edge regions in the single Aβ fibril occurred with additional KPFM scans (from scan #1 to #13 in Figure 4a). Also, the generated surface potential was confined to a very narrow outer region of the thickness of the single Aβ fibril, as was the distribution of its lateral piezoelectric vectors we observed.

We analyzed representative transverse profiles for the single Aβ fibril's height and surface potential from the KPFM images with different scanning numbers. As shown in Figure 4b and Figure S7 (Supporting Information), the height profiles of the single Aβ fibril exhibited an arch‐shape with a maximum height of ≈5 nm over a transverse width of ≈50 nm, and negligible changes even during the additional 12 scans of continuous sideband KPFM measurements based on elasticity derived from multiple hydrogen bonds. The matching trace and retrace of Aβ fibril's KPFM height images suggest the absence of artifacts (Figure S8, Supporting Information). Note that the exaggerated transverse width of the thread‐like Aβ fibril is a result of the tip–sample convolution and was not an error. In contrast to the height profile, the surface potential profile of the Aβ fibril showed the occurrence of two prominent peaks reaching 2,233 mV (from 491 mV, Δ = +1,742 mV) and 915 mV (from 486 mV, Δ = +429 mV) by the last scan (Figure 4c). This surface potential increment of the single Aβ fibril (Δ > +1700 mV) is high above that of an ideal crystalline collagen fiber (from +10 to +100 mV),^[^ ^56^, ^57^, ^58^ ^]^ which is the thermodynamically‐stable and major component of normal tissues in human body. Moreover, we found that the Aβ fibril can release the accumulated surface charges to the surroundings in response to applied electrostatic forces (Figure S9, Supporting Information). These surface potential behaviors of the piezoelectric Aβ fibril occurred in a very narrow region (only 2.9 nm out of 50 nm transverse width in the KPFM profile). Our nanoindentation and oscilloscope analysis results of Aβ fibrils further supported these KPFM analysis results by directly displaying the mechanical pressure‐induced voltage generation (Figures S10 and S11, Supporting Information).

## 3 Discussion

We infer the lateral piezoelectricity of Aβ fibrils to have originated from their molecular structure. According to the electron density map of Aβ fibrils,^[^ ^25^, ^26^ ^]^ the two Aβ peptides that make up the cross‐section form an identical LS‐shaped conformation (L‐shaped N‐terminal, S‐shaped C‐terminal), facing each other at a tilting angle of ≈10° relative to the longitudinal axis. Also, the Aβ peptides in Aβ fibrils comprise different hydrophobic clusters of adjacent amino acid residues (e.g., Leu17–Ile31–Phe19, Ala30–Ile32–Met35–Val40), which are stacked along the uniaxial direction.^[^ ^25^, ^26^ ^]^ As a result, the molecular structure of Aβ fibrils exhibits not only multiple crystallized amino acid residues but also an approximate 2_1_ screw symmetry rather than a C_2_ symmetry.^[^ ^25^ ^]^ Note that crystallized amino acid residues can exhibit different piezoelectric behaviors depending on their alignment (Table S3, Supporting Information).^[^ ^37^, ^38^, ^40^ ^]^ We attribute the lateral piezoelectric behavior of Aβ fibrils to the helically‐aligned amino acid residues along the longitudinal axis that allow the unidirectional changes in permanent dipole moment, similar to collagen fibers.^[^ ^59^, ^60^ ^]^ Also, we ascribe the giant lateral piezoelectric constant (44.1 pC N^‐1^) of Aβ fibrils to their close‐packed and self‐assembled molecular structure, in contrast to collagen fibers with lower lateral piezoelectric constants (0.31 to 12.0 pC N^‐1^) as well as less structural density due to the D‐band derived from multiple hole zones.^[^ ^31^ ^]^ While supporting theoretical calculations regarding the amino acid residue clusters with the approximate 2_1_ screw symmetry need to be conducted in the future, the strong lateral compared to negligible vertical piezoelectric properties of Aβ fibrils we have uncovered align consistently with their molecular structure.

Additionally, the lateral piezoelectric features of Aβ fibrils provide a subtle hint to elucidate abnormal behaviors of brain cells. According to patch clamp electrophysiology studies,^[^ ^14^, ^15^, ^16^, ^17^, ^61^, ^62^ ^]^ the accumulation of Aβ fibrils in the microglia and astrocyte induces depolarization of the plasma membrane and hyperexcitability in the mechanosensitive and voltage‐gated ion channels. Our 2D lateral piezoelectric vector map has not only demonstrated lateral piezoelectricity of Aβ fibrils but also their spiral pattern with at least four different domains following gamma distributions. Moreover, our electric potential analysis results have shown that the surface of Aβ fibrils allows the accumulation of piezoelectric charges with a potential difference of more than +1700 mV and releases them in response to applied pressure. This is in direct contrast to collagen fibers which exhibit linearly alternating lateral piezoelectric responses only along the longitudinal axis,^[^ ^31^ ^]^ and allow the limited surface potential increment from +10 to +100 mV under mechanical stimuli.^[^ ^56^, ^57^, ^58^ ^]^ Note that collagen fiber is the major component of the extracellular matrix that makes up normal biological tissues. Given the threshold voltage of different voltage‐gated ion channels (e.g., ‐40 mV of Ca^2+^ channels, −55 mV of Na^+^ channels),^[^ ^63^ ^]^ the lateral piezoelectricity of Aβ fibrils implies a link for the Aβ‐induced depolarization and hyperexcitability of AD brains.^[^ ^19^ ^]^ Nonetheless, it is difficult to answer whether the piezoelectric behaviors of Aβ fibrils actually work in human brains since we have analyzed pure and synthetic Aβ fibrils in the absence of any brain components (e.g., lipids, tissue metabolites) that can affect the surface of Aβ fibrils. Also, our microscopic analyses were conducted on dried Aβ fibrils due to possible displacements (Figure S12, Supporting Information). Despite these limitations, we have revealed the piezoelectric nature of crystalline Aβ fibrils without any chemical modification to provide a clue to future AD pathologies.

In conclusion, we have unveiled the giant lateral piezoelectric characteristics and piezoelectricity‐associated surface potential behaviors of Aβ fibrils. Our vector PFM analysis results have revealed that the lateral piezoelectric constant of Aβ fibrils is 44.1 pC N^‐1^, which is at least 3.6 times higher than that of collagen fibers constituting normal biological tissues. Also, our continuous sideband KPFM scanning analysis results have shown that Aβ fibrils exhibit an extraordinary surface potential increment of +1742 mV, at least 17 times higher than that of collagen fibers, in response to external electrostatic pressures. We have found that these piezoelectricity‐associated behaviors of Aβ fibrils are concentrated at the edges where they contact with the surroundings. This study provides a small piece of the puzzle in AD pathology by unveiling the piezoelectric nature of Aβ fibrils, the pathological hallmark that leads to the onset of neurogenerative symptoms.

## 4 Experimental Section

### Preparation of Aβ Fibrils

Monomeric Aβ (1‐42; human) peptide was obtained from Anaspec (CA, USA). Mature Aβ fibrils were prepared through the self‐assembly of Aβ peptides following the literature.^[^ ^64^, ^65^, ^66^, ^67^ ^]^ Monomeric Aβ peptides were completely dissolved in a basic aqueous solution (250 mM of sodium hydroxide, 0.3 mM of sodium carbonate, 0.3 mM of acetonitrile) with concentration of 400 µM. As‐prepared Aβ monomer solution was diluted with a phosphate buffer (8.5 mM of sodium phosphate, 8.5 mM of sodium chloride, 14 µM of sodium hydroxide, 0.85 mM of sodium hydroxide, 6.0 vol% of acetonitrile, pH 7.4) with the final concentration of 40 µM. Afterward, Aβ monomer solution was incubated for 48 h at 37 °C to obtain mature Aβ fibrils.

### Analysis of Aβ Fibrils

2D XRD pattern of Aβ fibrils was collected by using a small‐angle X‐ray scattering instrument (NANOPIX, Rigaku, Japan) after lyophilization. 1D XRD profiles of Aβ fibrils were obtained by analyzing the 2D XRD pattern. CD spectrum of Aβ fibrils was gathered by using a CD spectropolarimeter (Jasco‐815 150‐L, Jasco, Japan) under nitrogen atmosphere. Vertical and lateral PFM images of Aβ fibrils were obtained by a scanning probe microscope (Cypher‐ES, Asylum Research, CA, USA) with a platinum/iridium‐coated silicon cantilever (CONTPt, Nanoworld, Switzerland). The scanning angle of the PFM cantilever was set to zero,^[^ ^68^, ^69^ ^]^ with scan rate of 0.5 Hz and loading force of 10 nN.^[^ ^46^, ^70^, ^71^ ^]^ This setup minimized electrostatic contributions to the torsional movement of the tip, allowing measurements to focus on the shear electromechanical effects of Aβ fibrils. Vertical and lateral drive frequencies were 53 kHz and 309 kHz, respectively. Collected vertical and lateral PFM images were analyzed by using *IGOR Pro* software (Wavemetrics, OR, USA) and Python codes.^[^ ^72^, ^73^, ^74^ ^]^ Lateral piezoelectric vector map was obtained by merging the individual pixel information for the same spots on one lateral piezoresponse image of a single Aβ fibril and another lateral piezoresponse image collected after a 90° rotation on the microscope sample stage. Measurements in this study are presented in direct piezoelectric units of pC N^‐1^, which are thermodynamically equiv. to the inverse piezoelectric units of pm V^‐1^. Continuous sideband KPFM images were obtained by a scanning probe microscope (NX10, Park Systems, Republic of Korea) with a platinum/iridium‐coated silicon cantilever (PPP‐EFM, Nanosensors, Switzerland) under ambient condition (scan rate: 1 Hz). Frequency modulation mode with a single‐pass method was used for sideband KPFM measurement. The work function of the probe was calibrated using a freshly cleaved highly ordered pyrolytic graphite (HOPG) reference sample (Φ_tip_ = 5.65 eV). Collected KPFM images were analyzed by using the *XEI* software (Park Systems, Republic of Korea). Prior to the microscopy investigations, Aβ fibrils were coated on a cleaned Au/Cr‐coated silicon wafer without any chemical modification to reveal inherent piezoelectric nature and allowed to dry completely. All analyses were performed through multiple and reproducible Aβ fibril preparations.

### Statistical Analysis

Statistical analysis of lateral piezoelectric vectors was conducted by Excel 2019 (Microsoft, WA, USA). Comparisons were performed with one‐way analysis of variances (ANOVA). A *P*‐value less than 0.05 was considered statistically significant.

## Conflict of Interest

The authors declare no conflict of interest.

## Author Contributions

J.J. and S.J. contributed equally to this work. J.J. and S.J. designed and conducted this study. C.B.P. conceived of the initial idea. J.J. developed the initial idea and planned figures. S.H. and C.B.P. supervised this study. J.J. and S.J. wrote the manuscript. J.J., S.J., S.H., and C.B.P. edited the manuscript. All authors contributed to the interpretation of the results.

## Supporting information

Supporting Information

## Data Availability

The data that support the findings of this study are available from the corresponding author upon reasonable request.
