# Supplementary material for: Lateral Piezoelectricity of Alzheimer's Aβ Aggregates
Source: Adv Sci (Weinh). 2024 Aug 19;11(39):2406678. doi: 10.1002/advs.202406678 (PMC11497015; doi:10.1002/advs.202406678)
Supplement: Supplementary file 1 — Supporting Information [file ADVS-11-2406678-s001.pdf]

ADVANCED SCIENCE
Open Access

Supporting Information

for *Adv. Sci.*, DOI 10.1002/advs.202406678

Lateral Piezoelectricity of Alzheimer's A*β* Aggregates

Jinhyeong Jang, Soyun Joo, Jiwon Yeom, Yonghan Jo, Jingshu Zhang, Seungbum Hong and Chan Beum Park**

Supporting Information

Lateral Piezoelectricity of Alzheimer's Aβ Aggregates

*Jinhyeong Jang [a, b] †, Soyun Joo [a] †, Jiwon Yeom [a], Yonghan Jo [a], Jingshu Zhang [a], Seungbum Hong [a] *, Chan Beum Park [a] **

[a] Department of Materials Science and Engineering, Korea Advanced Institute of Science and Technology (KAIST), 335 Science Road, Daejeon 34141, Republic of Korea

[b] Applied Science Research Institute, Korea Advanced Institute of Science and Technology (KAIST), 335 Science Road, Daejeon 34141, Republic of Korea

† These authors contributed equally to this work.

* E-mail: seungbum@kaist.ac.kr (S. Hong); parkcb@kaist.ac.kr (C. B. Park)

Supporting Calculations

Calculation 1. Hertzian model about possible deformation during vector PFM measurement

Calculation 2. Coulomb's law about possible charge noise during vector PFM measurement

Supporting Figures

Figure S1: TEM and AFM topography images of multiple entangled Aβ fibrils

Figure S2: CD spectra and BeStSel analysis results of Aβ monomers and fibrils

Figure S3: Additional vertical and lateral PFM images of different Aβ fibrils

Figure S4: Additional profiles from lateral PFM images of different Aβ fibrils

Figure S5: Trace and retrace of Aβ fibril's lateral piezoelectric images at two different angles

Figure S6: Scheme for lateral piezoresponse magnitude.

Figure S7: Multiple topographic AFM and KPFM scanning images of a single Aβ fibril

Figure S8: Representative trace and retrace of Aβ fibril's KPFM images

Figure S9: Additional analysis results of continuous KPFM images to show surface potential decrement of a single Aβ fibril under applied electrostatic forces

Figure S10: Nanoindentation test results of Aβ fibrils

Figure S11: Oscilloscope analysis result of Aβ fibrils

Figure S12. Schematic illustration for PFM analysis on Aβ fibrils in dried environment

Supporting Tables

Table S1: Tip specifications of different AFM studies on various biological components

Table S2: Lateral piezoelectric constants of collagen fibers

Table S3. Piezoelectric constants of normal biological materials

Supplementary Calculation 1.

Hertzian model about possible deformation during vector PFM measurement

Based on the Hertzian model, we provide the following calculations to verify that the Aβ fibrils did not undergo significant deformation during our PFM measurements:

$$F = \frac{4E_{tip}E_{A\beta}}{3(1 - \nu^2)(E_{tip} + E_{A\beta})}(\Delta z_{sample})^{3/2}\sqrt{r}$$

where F is the force acting on the tip (10 nN), E_{tip} and $E_{A\beta}$ are the elastic moduli of the tip (PtIr; 200 GPa) and the Aβ fibrils (3.2 GPa, *please see our* ***Supplementary Figure S6-e***), respectively, ν is the Poisson's ratio of Aβ fibrils (biological materials typically fall in the range of 0.2 to 0.5; we used 0.25), Δz_{sample} is the indentation depth, and r is the tip radius (25 nm, *please see our* ***Supplementary Table S1***). Then, the calculated Δz_{sample} becomes 0.125 nm. Thus, for the PFM measurements, Aβ fibrils underwent an indentation of 0.125 nm, which is orders of magnitude smaller than their actual dimensions. We can safely assume that Aβ fibrils did not significantly deform.

Supplementary Calculation 2.

Coulomb's law about possible charge noise during vector PFM measurement

Based on the Coulomb's law, we consider the amount of electric charge (q) which is generated during PFM measurements by multiplying the applied force F (10 nN) with the measured lateral piezoelectric coefficient (44.1 pC/N), yielding 440×10^{-21} C. Then, we can calculate the electric force acting on the tip due to the generated charge (direct piezoelectric effect):

$$F_{electric} = qE$$

The maximum field (E) can be estimated from the magnitude of the AC drive voltage (1 V) and the dimension of Aβ fibrils (10 nm) to be 10^8 V/m. Thus, $F_{electric}$ becomes 44 pN. We can safely assume that the direct piezoelectric effect from the Aβ fibrils lateral piezoelectricity would not significantly affect the magnitudes of piezoresponse recorded in our PFM measurements.

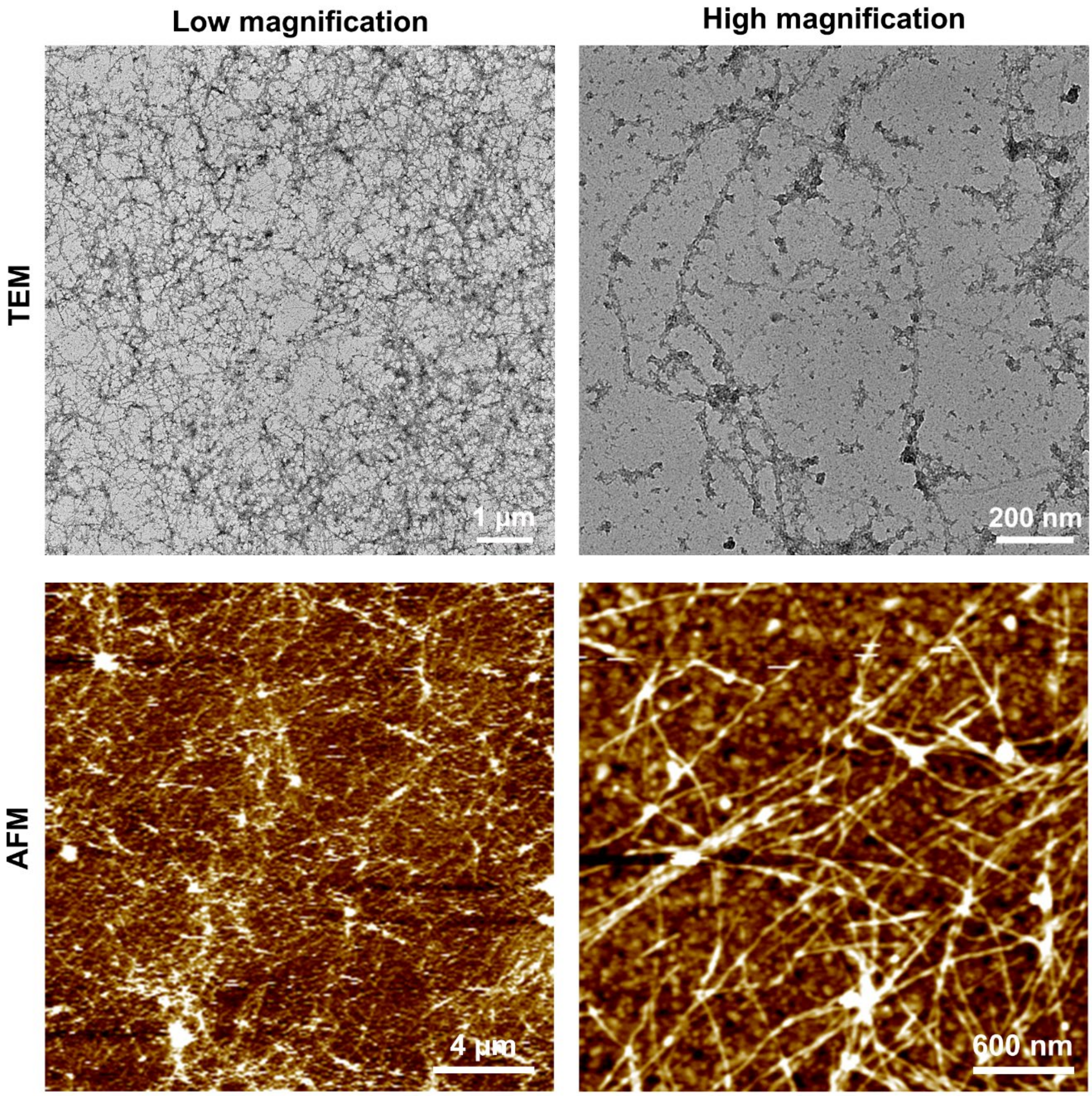

Figure S1. Transition electron microscopy (TEM) and AFM topography images of multiple entangled Aβ fibrils in low and high magnifications. The self-assembly process of monomeric Aβ peptides forms thread-like insoluble Aβ fibrils, which are physically entangled with each other to construct aggregated network structures on plasma membranes in AD brains. TEM images of Aβ fibrils were acquired after staining with UranyLess EM Stain (Electron Microscopy Sciences Inc., PA, USA). AFM images of Aβ fibrils were collected under ambient conditions without any staining.

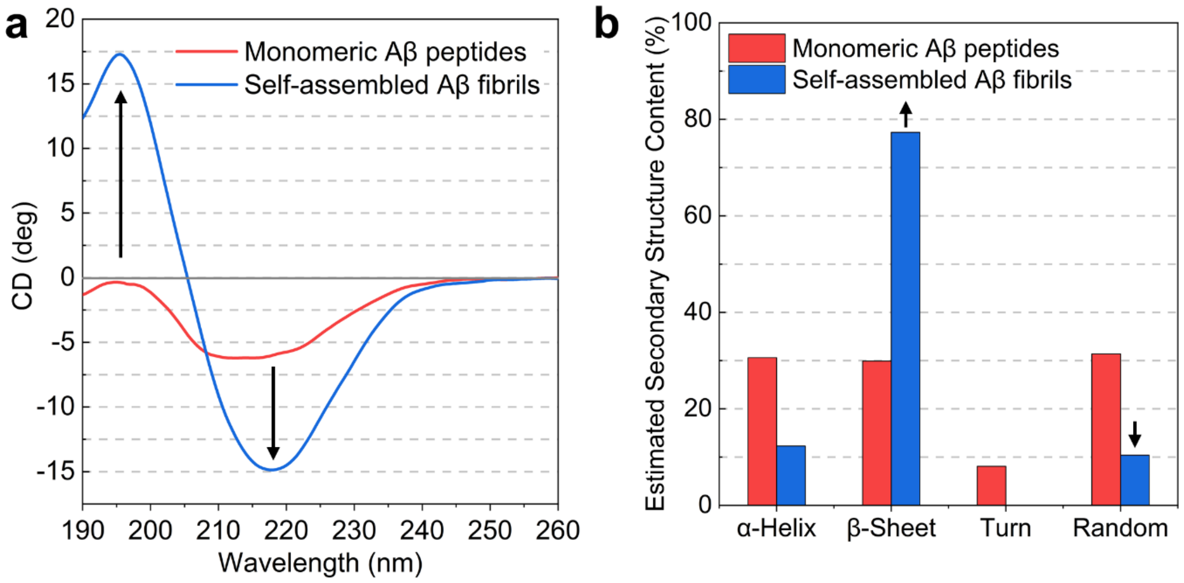

Figure S2. Circular dichroism (CD) analysis results of monomeric Aβ peptides and self-assembled Aβ fibrils. (a) CD spectra show that self-assembled Aβ fibrils have a dominant β-sheet protein secondary structure based on the occurrence of two characteristic peaks at 196 and 218 nm. It is contrast to monomeric Aβ peptides with dominant random secondary structure. (b) Quantitative BeStSel (Beta Structure Selection) analysis results performed based on these CD spectra also demonstrate that the transition from monomeric Aβ peptides to self-assembled Aβ fibrils leads to a noticeable increase in β-sheet protein secondary structure (from 29.9% to 77.3%) and decrease in random structure (from 31.4% to 10.4%). BeStSel analysis of protein secondary structures was conducted through a web server (https://bestsel.elte.hu) operated based on the literature.[1-3]

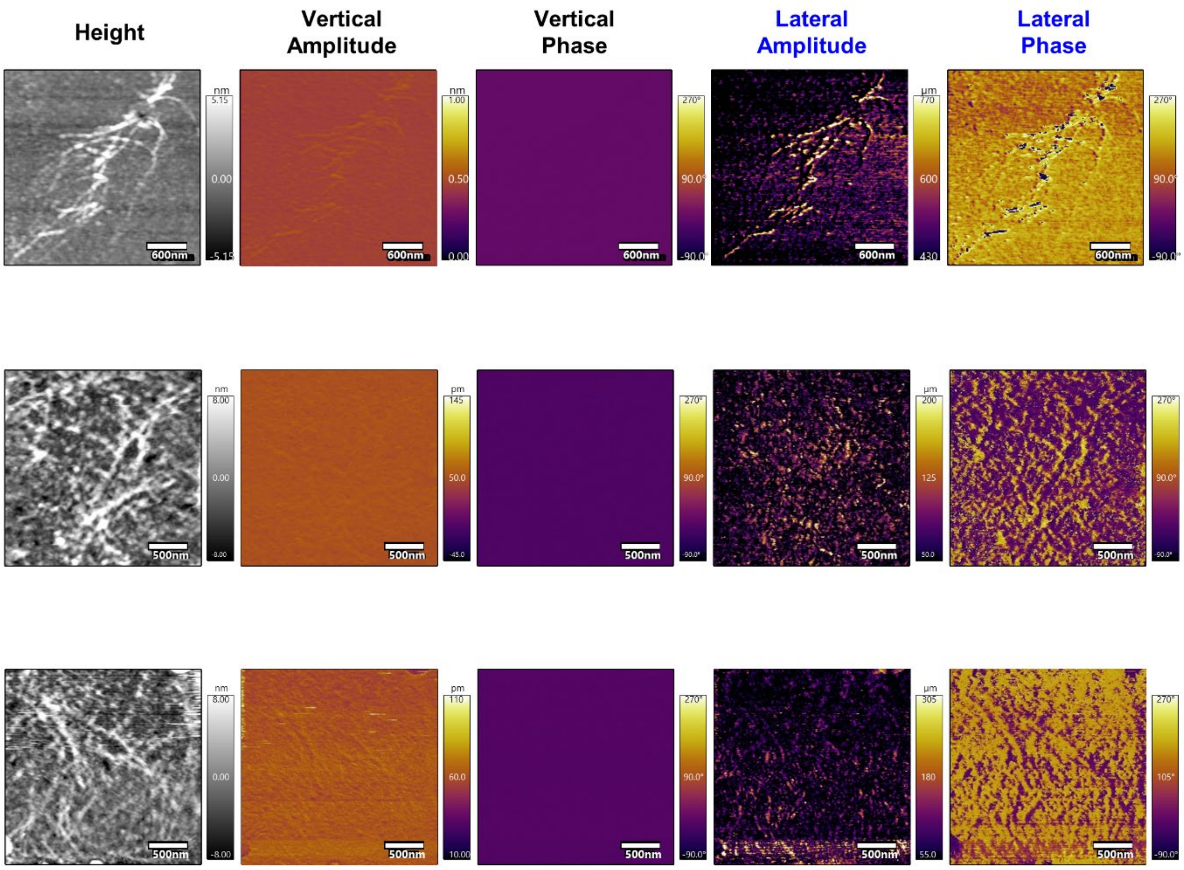

Figure S3. Additional vertical and lateral PFM images of different Aβ fibrils. Each set of Aβ fibrils was independently prepared and imaged at different times. These PFM images demonstrate multiple and reproducible Aβ fibril preparations of this study.

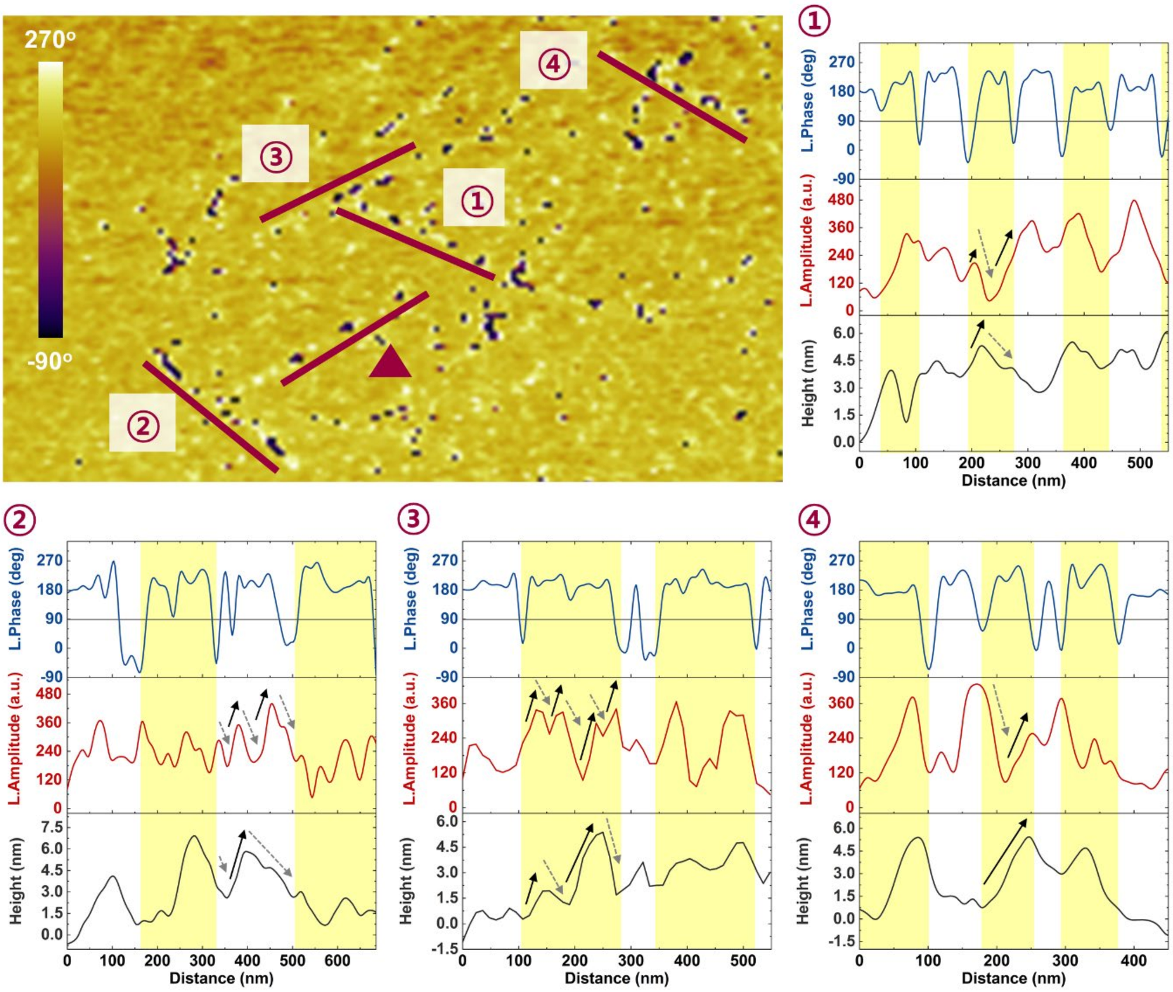

Figure S4. Additional profiles of different Aβ fibrils with their lateral PFM phases, lateral PFM amplitudes, and topographic heights. The lateral phase profiles of different Aβ fibrils do not match the height profiles and do not show a regular pattern. These results indicate that the lateral piezoelectric signals are derived from the nature of Aβ fibrils rather than the shot noise and topographic cross-talk. Each profile displays information for a single line without averaging for multiple pixels. A single line marked with a red triangle is shown in *Main Figure*.

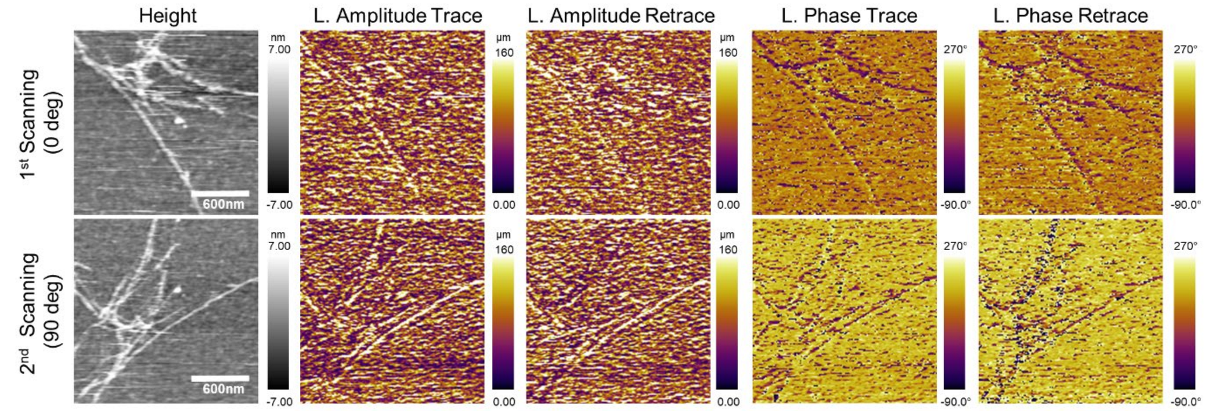

Figure S5. Trace and retrace of Aβ fibril's lateral piezoelectric images taken at two different angles. The second line represents the results from scanning the same area as the first line after physically rotating the sample by 90 °. As shown in the images, the trace and retrace are almost identical at two different angles, suggesting the absence of artifacts.

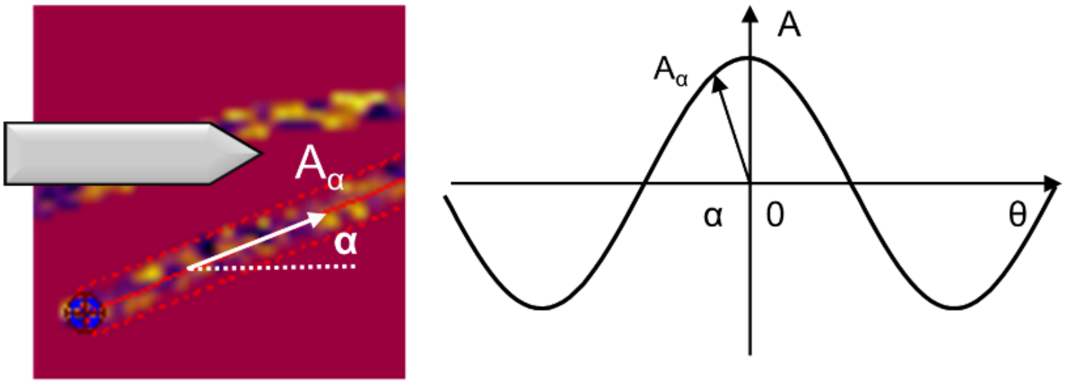

Figure S6. Scheme for extraction of maximum lateral piezoresponse magnitude that varies sinusoidally with fibril alignment orientation angles. Here, α represents the angle between the scanning direction – which is parallel to the cantilever length – and the imaged fibril, while A_α represents the maximum lateral piezoresponse magnitude. The distribution of A_α as a function of α was fitted to a cosine function and calibrated against a standard $LiNbO_3$ reference sample, which was physically rotated to vary its in-plane piezoelectric alignment relative to the AFM cantilever.

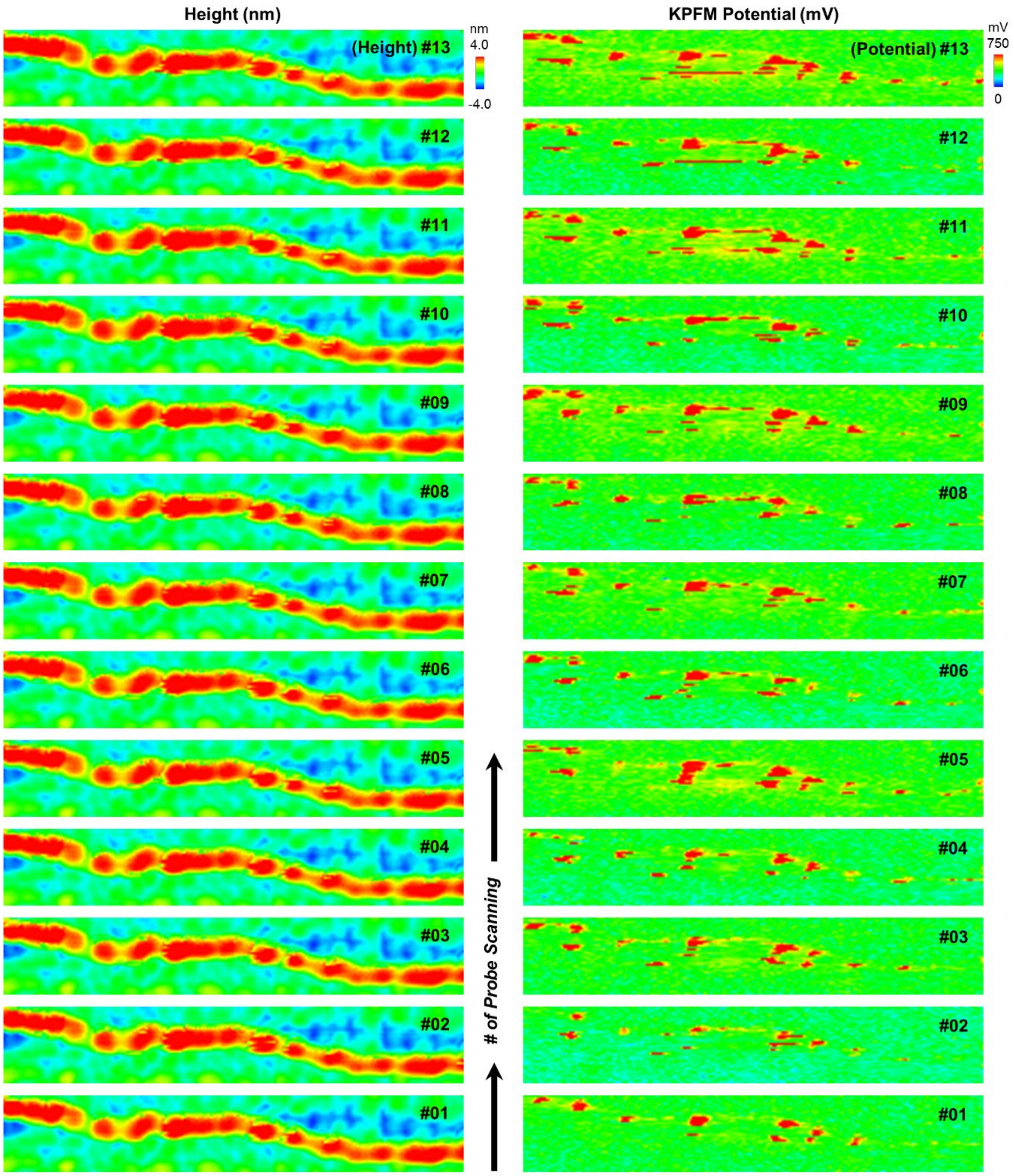

Figure S7. Multiple height topography and KFPM potential images of a single Aβ fibril collected by continuous KPFM scanning. As shown in the height topography images of single Aβ fibrils with different scan numbers, continuous KPFM scanning with non-contact mode did not cause any significant deformation of the single Aβ fibril.

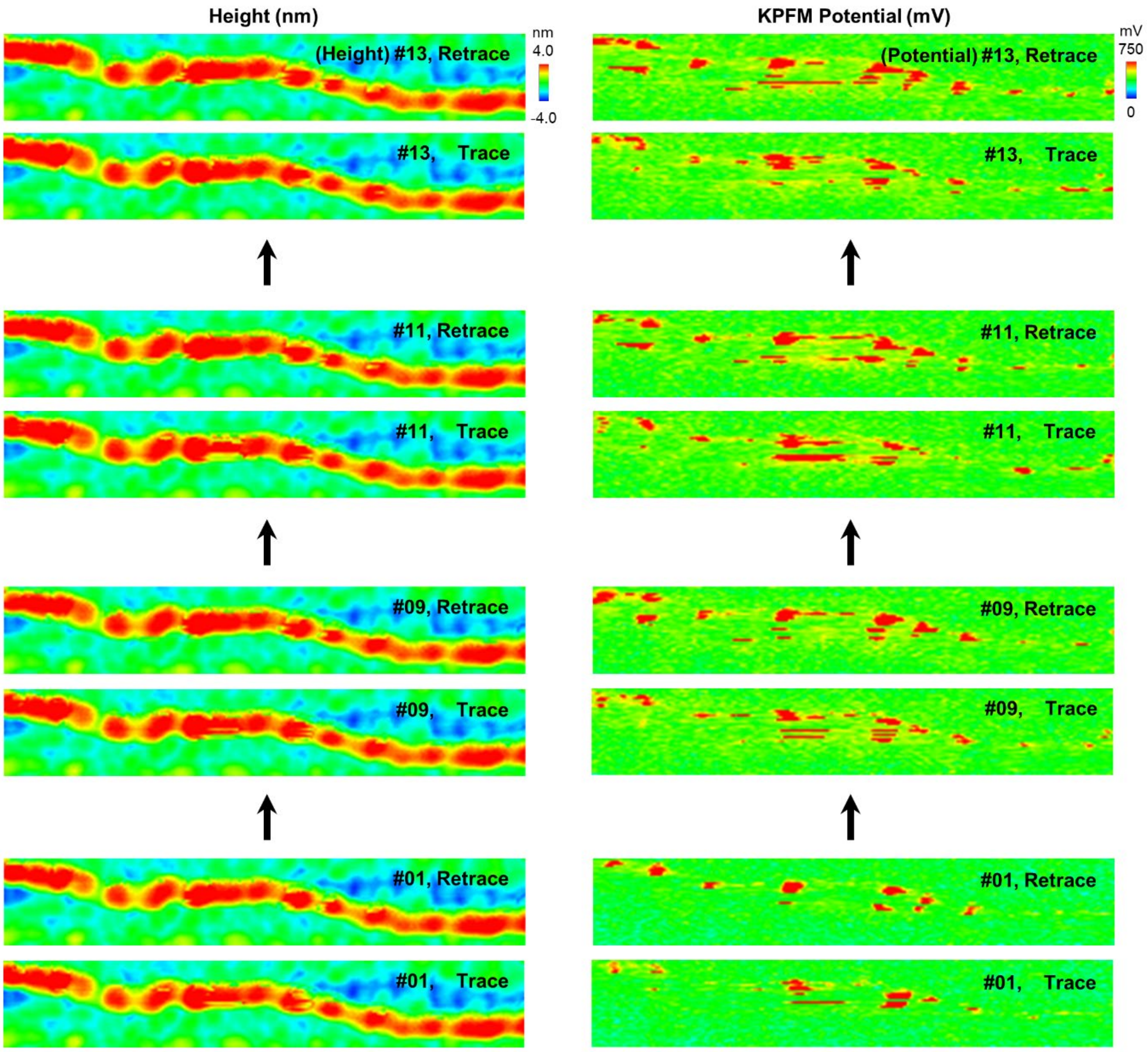

Figure S8. Representative trace and retrace of Aβ fibril's KPFM images. As shown in the images, trace and retrace of Aβ fibril's KPFM height images are almost identical. Thus, the surface profile was accurately tracked in these measurements, suggesting the absence of artifacts during KPFM images. Note that the mismatching trace and retrace of Aβ fibril's KFPM potential images are derived from augmenting effects of applied electrostatic forces by the AFM tip. The difference between trace and retrace potential images further suggest the piezoelectricity-derived, charge accumulative characteristics of Aβ fibrils.

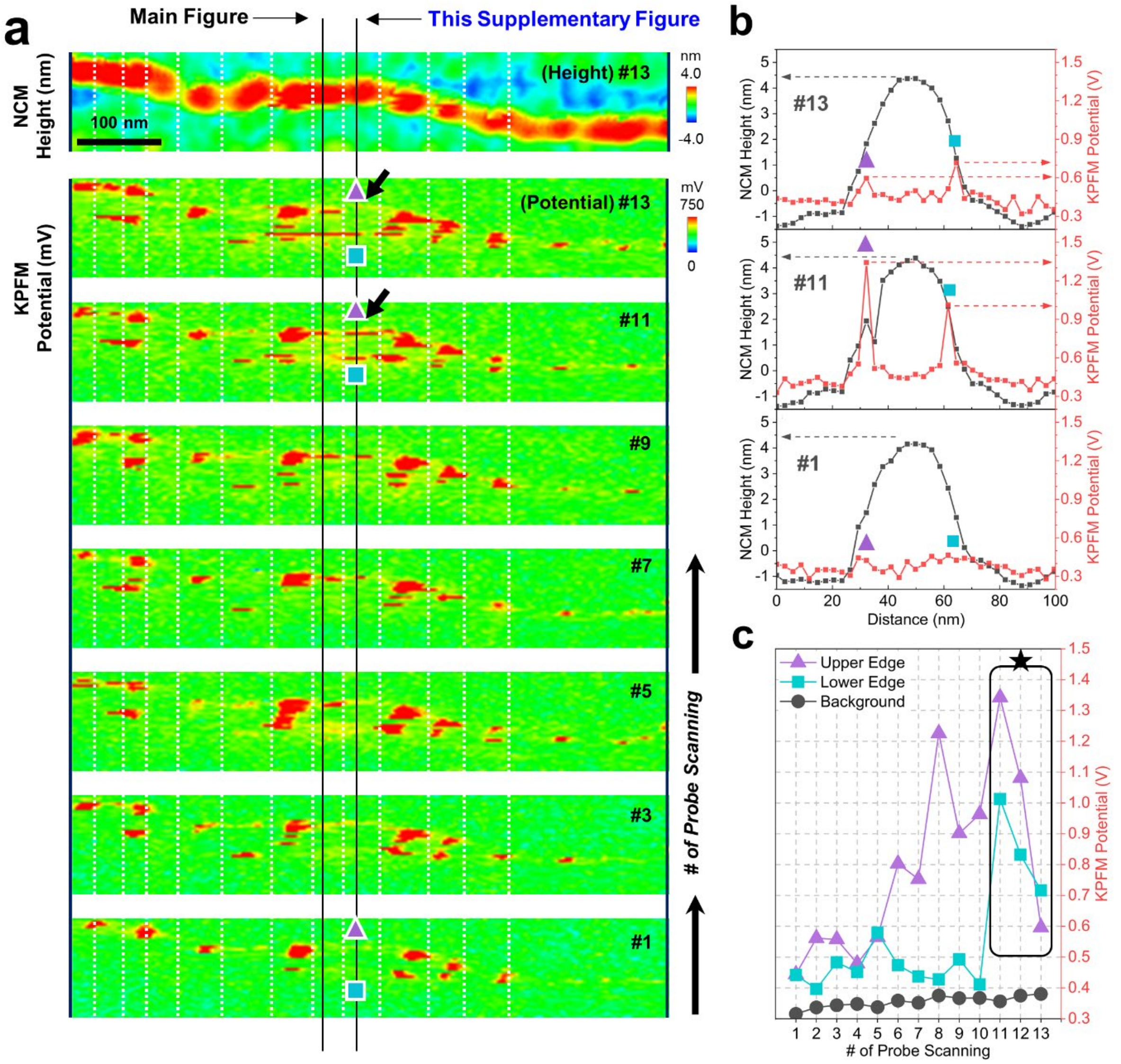

Figure S9. Surface potential decrement on a single Aβ fibril under repetitively-applied electrostatic pressures. (a) Continuous KPFM images of a single Aβ fibril at different scanning numbers. Vertical black lines mean the analysis sites in the single Aβ fibril. Purple triangle and cyan square in the KPFM images mark two peak sites, respectively. (b) The representative surface potential and height profiles for showing surface potential decrement on the single Aβ fibril under applied electrostatic stress. (c) Surface potential decrement following the increment of scanning numbers from 11 to 13. This range is highlighted by square box with a black star in the figure.

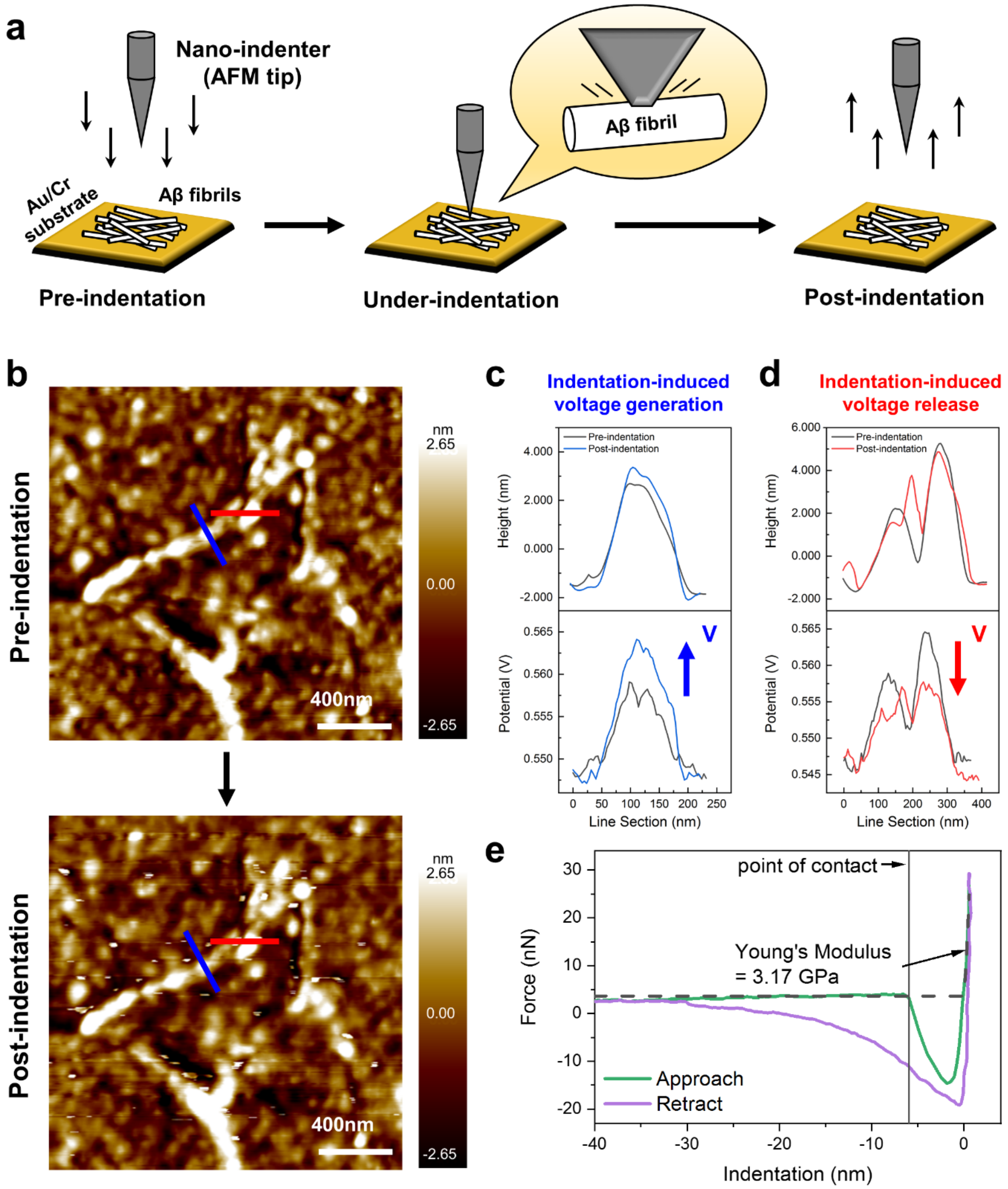

Figure S10. Nanoindentation test results of Aβ fibrils. Direct piezoelectric effects are tested by measuring the surface potential change before and after nano-indentation of Aβ fibrils. (a) Schematic illustration of the nanoindentation test for Aβ fibrils coated on an Au/Cr substrate. (b) Topography of Aβ fibrils acquired before and after applying nanoindentation. The post-indentation image shows white spots resulting from physical interactions between the tip and the sample, while the approximate structure of the Aβ fibril remains intact. Height and potential line sections obtained from the (c) blue and (d) red lines are shown in the topography of Aβ fibrils. The height remains unchanged, while electrical voltage increment

and decrement were observed at the blue and red lines, respectively. (d) During the nanoindentation test, Young's modulus of Aβ-fibrils was measured to be 3.17 GPa, closely approximating the literature [Nanoscale 4, 4426-4429 (2012)]. Both KPFM and nano-indentation measurements were performed using a chemically inert conductive diamond tip (CDT-FMR, NanoWorld, Switzerland). Collected force curves were analyzed using the Hertz model to estimate the elastic moduli.

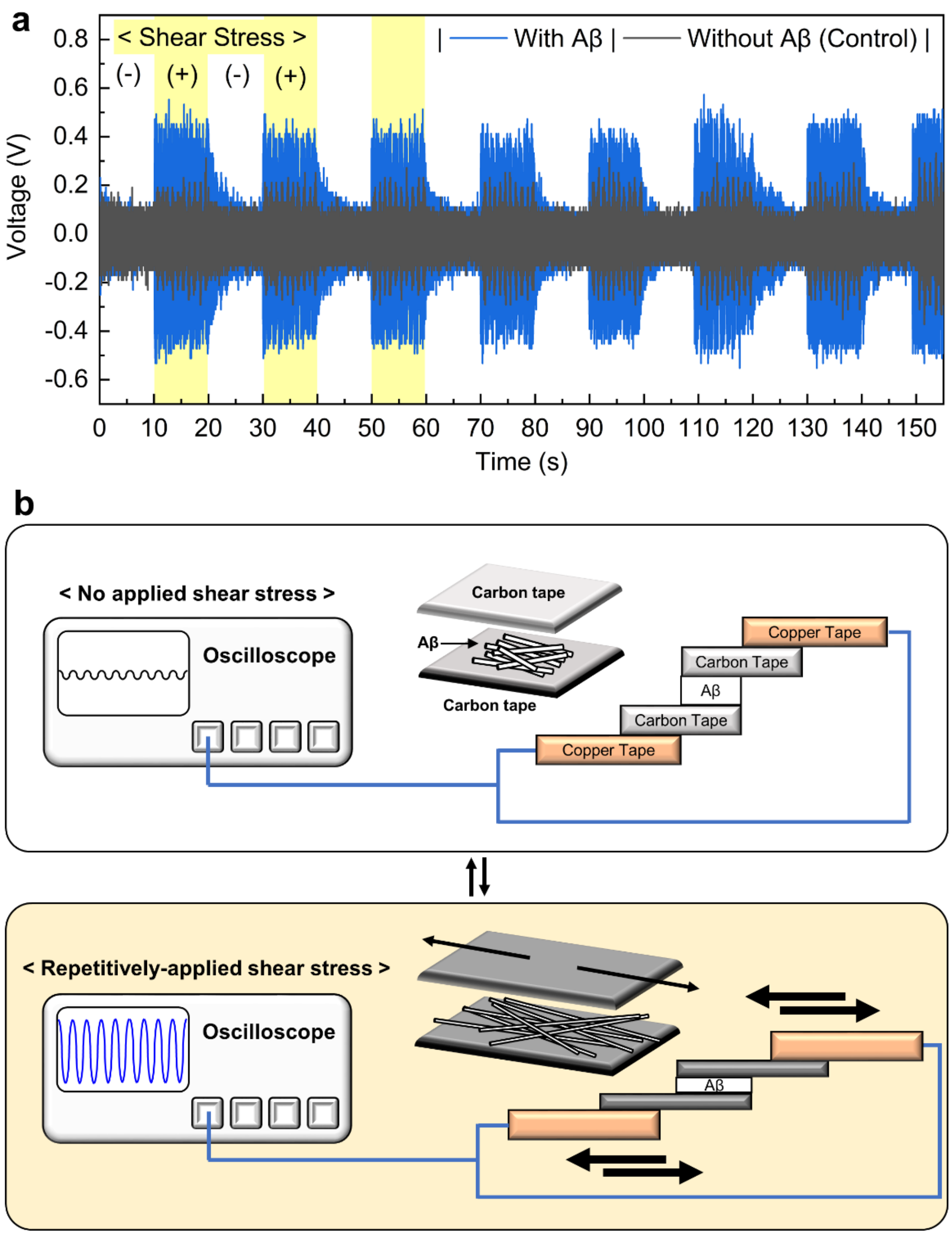

Figure S11. Lateral piezoelectricity of bulk Aβ fibrils. (a) Oscilloscope analysis results of Aβ fibrils subjected to repetitively applied shear stress with the duration of 10 seconds. (b) Schematic illustration of experimental setup. Lyophilized Aβ fibril powders were placed between two adhesive and stretchable carbon tapes, which were connected to non-stretchable adhesive copper tapes interfaced with an oscilloscope. When shear stress was applied to the copper tapes, shear strain was induced in both the carbon tape and attached Aβ fibrils, resulting in the Aβ fibril's lateral piezoelectric response. As shown in the oscilloscope analysis results, the lateral piezoelectricity of Aβ fibrils was dependent on the applied shear stress.

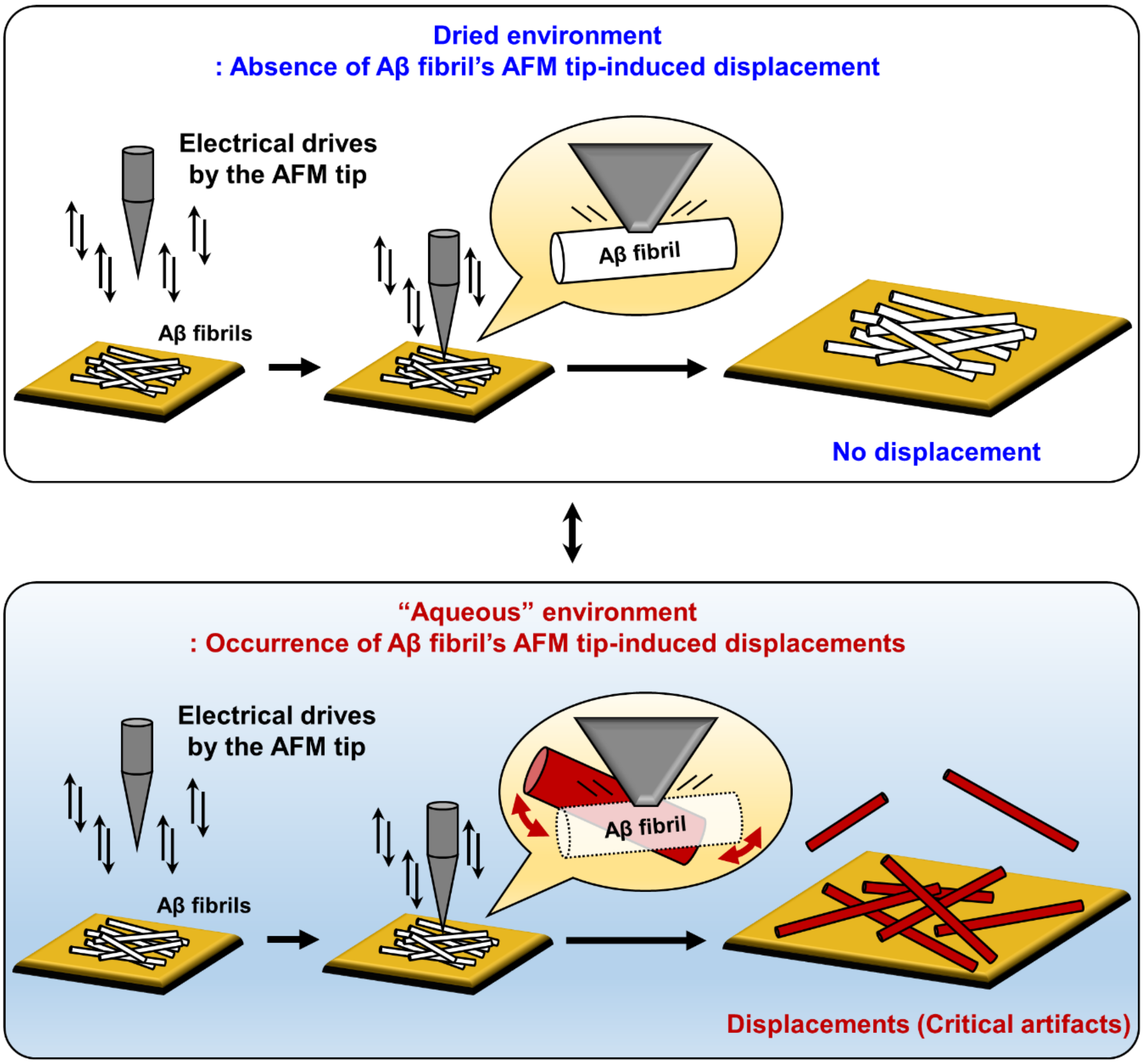

Figure S12. Schematic illustration for PFM analysis on Aβ fibrils in dried environment. Vector PFM analyses on Aβ fibrils were conducted in a dried condition. If these analyses are conducted in an aqueous environment, the displacement of helical-structured and very thin Aβ fibrils should occur due to the applied electrical drives by the AFM probe tip. These displacements act as significant artifacts, and interfere with the reliability of analysis results. Note that we analyzed Aβ fibrils without any chemical modification, such as covalent bond formation between Aβ fibril and conductive substrate to prevent tip-induced displacements, for revealing their inherent nature.

Table S1. Tip specifications of different AFM studies on various biological components reported from different literature.

Material	Probe (Manufacturer)	Nominal Spring Constant (N m^{-1})	Tip Radius (nm)	Tip Shape	Reference Number	Published Year
Aβ fibrils	CONTPt, for Vector PFM (Maker:Nanoworld)	0.2	< 25	Square pyramidal	This Study	
	PPP-EFM, for Sideband KPFM (Maker: Nanosensors)	2.8		Square pyramidal		
Type I collagen fiber	Not explicitly mentioned (Maker: MikroMasch)	0.15	Unknown	Unknown	[4]	2009
Non-pathological insulin fibrils	RC800PB (tip 2) (Maker: Olympus)	0.06	42	Square pyramidal	[5]	2010
Di-peptide (Phe-Phe)	Not explicitly mentioned	0.02−1	Unknown	Unknown	[6]	2010
Tri-peptides (Pro-Phe-Phe & Hyp-Phe-Phe)	RTESPA 525 (Maker: Bruker)	200	8	Square pyramidal	[7]	2021
Collagen fiber	PPP-EFM (Maker: Nanosensors)	2.8	25	Square pyramidal	[8]	2017
Type I collagen fiber membrane	DPE-18 (Maker: MikroMasch)	3.5	40	Unknown	[9]	2014
Type I collagen fiber	Not explicitly mentioned (Maker: MikroMasch)	0.15	Unknown	Unknown	[10]	2009
Type II collagen fiber	CSC37 (tip B) (Maker: MikroMasch)	0.3	8	Square pyramidal	[11]	2014
Type I collagen fiber	3XC-GG (tip 1) (Maker: OPUS)	0.3	30	Tetrahedral	[12]	2020
Wild type collagen fiber	3XC-GG (tip 3) (Maker: OPUS)	2.5	30	Tetrahedral	[13]	2022

Table S2. Lateral piezoelectric constants of collagen reported from different literature.

Collagen Types	Lateral Piezoelectric Constant (pC N^{-1})	Reference Number	Published Year
Collagen fiber (extracted from 4-week old rat tendon)	12.0	[14]	2017
Collagen fiber (extracted from bovine Achilles tendon)	4.2	[15]	1964
Collagen fiber (molecular dynamics simulation)	2.6	[16]	2016
Collagen fiber (extracted from bovine Achilles tendon)	2	[17]	1975
Type I collagen fiber (extracted from bovine Achilles tendon)	2	[4]	2009
Collagen fiber (extracted from horse Achilles tendon)	1.6	[15]	1964
Type I collagen fiber membrane	1.6	[18]	2014
Type I collagen fiber (extracted from bovine Achilles tendon)	1	[10]	2009
Type II collagen fiber (extracted from chicken sternum cartilage)	0.7	[19]	2014
Type I collagen fiber (extracted from bovine Achilles tendon)	0.51	[12]	2020
Wild type collagen fiber (extracted from mouse bone)	0.31	[13]	2022

Table S3. Piezoelectric constants of normal amino acid, peptide, and protein aggregates, referred to the literature.[20]

Assemblies	Type	Piezoelectric Constant (pC N^{-1})	Reference Number	Published Year
Alzheimer's Aβ fibrils	Pathological hallmark	44.1	This study	
Insulin hormone protein	Normal biological materials with crystallization process in ideal conditions	Numeric value is not available	[5]	2010
Keratin protein		1.8	[21]	2000
Lysozyme protein		6.5	[22]	2017
Collagen protein		Please see **Table S2**	-	-
Glycine	Artificial amino acid/peptide crystals	178	[23]	2018
Asparagine		13		
Leucine		12.5		
Histidine		18		
Methionine		15		
Aspartate		13		
Isoleucine		25		
Cysteine		11.4		
Alanine		17.75	[24]	2019
Diphenylalanine		60	[6]	2010
poly-γ-benzyl-l-glutamate		25	[25]	2011
poly-γ-methyl-l-glutamate		2	[21]	2000

Supporting References

1. Micsonai, A.; Wien, F.; Kernya, L.; Lee, Y.-H.; Goto, Y.; Réfrégiers, M.; Kardos, J., *Proc. Natl. Acad. Sci. U.S.A.* **2015,** *112* (24), E3095-E3103.
2. Micsonai, A.; Wien, F.; Bulyáki, É.; Kun, J.; Moussong, É.; Lee, Y.-H.; Goto, Y.; Réfrégiers, M.; Kardos, J., *Nucleic Acids Res.* **2018,** *46* (W1), W315-W322.
3. Micsonai, A.; Moussong, É.; Wien, F.; Boros, E.; Vadászi, H.; Murvai, N.; Lee, Y.-H.; Molnár, T.; Réfrégiers, M.; Goto, Y.; Tantos, Á.; Kardos, J., *Nucleic Acids Res.* **2022,** *50* (W1), W90-W98.
4. Minary-Jolandan, M.; Yu, M.-F., *ACS Nano* **2009,** *3* (7), 1859-1863.
5. Nikiforov, M. P.; Thompson, G. L.; Reukov, V. V.; Jesse, S.; Guo, S.; Rodriguez, B. J.; Seal, K.; Vertegel, A. A.; Kalinin, S. V., *ACS Nano* **2010,** *4* (2), 689-698.
6. Kholkin, A.; Amdursky, N.; Bdikin, I.; Gazit, E.; Rosenman, G., *ACS Nano* **2010,** *4* (2), 610-614.
7. Bera, S.; Guerin, S.; Yuan, H.; O'Donnell, J.; Reynolds, N. P.; Maraba, O.; Ji, W.; Shimon, L. J. W.; Cazade, P.-A.; Tofail, S. A. M.; Thompson, D.; Yang, R.; Gazit, E., *Nat. Commun.* **2021,** *12* (1), 2634.
8. Denning, D.; Kilpatrick, J. I.; Fukada, E.; Zhang, N.; Habelitz, S.; Fertala, A.; Gilchrist, M. D.; Zhang, Y.; Tofail, S. A. M.; Rodriguez, B. J., *ACS Biomater. Sci. Eng.* **2017,** *3* (6), 929-935.
9. Denning, D.; Paukshto, M. V.; Habelitz, S.; Rodriguez, B. J., *J. Biomed. Mater. Res. Part B* **2014,** *102* (2), 284-292.
10. Minary-Jolandan, M.; Yu, M.-F., *Nanotechnology* **2009,** *20* (8), 085706.
11. Denning, D.; Kilpatrick, J. I.; Hsu, T.; Habelitz, S.; Fertala, A.; Rodriguez, B. J., *J. Appl. Phys.* **2014,** *116* (6).
12. Kwon, J.; Cho, H., *ACS Biomater. Sci. Eng.* **2020,** *6* (12), 6680-6689.

13. Kwon, J.; Cho, H., *Commun. Biol.* **2022,** *5* (1), 1229.

14. Denning, D.; Kilpatrick, J. I.; Fukada, E.; Zhang, N.; Habelitz, S.; Fertala, A.; Gilchrist, M. D.; Zhang, Y.; Tofail, S. A.; Rodriguez, B. J., *ACS Biomater. Sci. Eng.* **2017,** *3* (6), 929-935.

15. Fukada, E.; Yasuda, I., *Jpn. J. Appl. Phys.* **1964,** *3* (2), 117.

16. Zhou, Z.; Qian, D.; Minary-Jolandan, M., *ACS Biomater. Sci. Eng.* **2016,** *2* (6), 929-936.

17. Netto, T. G.; Zimmerman, R. L., *Biophys. J.* **1975,** *15* (6), 573-576.

18. Denning, D.; Paukshto, M. V.; Habelitz, S.; Rodriguez, B. J., *J. Biomed. Mater. Res. Part B Appl. Biomater.* **2014,** *102* (2), 284-292.

19. Denning, D.; Kilpatrick, J.; Hsu, T.; Habelitz, S.; Fertala, A.; Rodriguez, B., *J. Appl. Phys.* **2014,** *116* (6), 066818.

20. Kim, D.; Han, S. A.; Kim, J. H.; Lee, J.-H.; Kim, S.-W.; Lee, S.-W., *Adv. Mater.* **2020,** *32* (14), 1906989.

21. Fukada, E., *IEEE Trans. Ultrason. Ferroelectr.* **2000,** *47* (6), 1277-1290.

22. Stapleton, A.; Noor, M. R.; Sweeney, J.; Casey, V.; Kholkin, A. L.; Silien, C.; Gandhi, A. A.; Soulimane, T.; Tofail, S. A. M., *Appl. Phys. Lett.* **2017,** *111* (14).

23. Guerin, S.; Stapleton, A.; Chovan, D.; Mouras, R.; Gleeson, M.; McKeown, C.; Noor, M. R.; Silien, C.; Rhen, F. M. F.; Kholkin, Andrei L.; Liu, N.; Soulimane, T.; Tofail, S. A. M.; Thompson, D., *Nat. Mater.* **2018,** *17* (2), 180-186.

24. Guerin, S.; O'Donnell, J.; Haq, E. U.; McKeown, C.; Silien, C.; Rhen, F. M.; Soulimane, T.; Tofail, S. A.; Thompson, D., *Phys. Rev. Lett.* **2019,** *122* (4), 047701.

25. Farrar, D.; Ren, K.; Cheng, D.; Kim, S.; Moon, W.; Wilson, W. L.; West, J. E.; Yu, S. M., *Adv. Mater.* **2011,** *23* (34), 3954-3958.
